# Reorganization of Cell Compartmentalization Induced by Stress

**DOI:** 10.3390/biom12101441

**Published:** 2022-10-08

**Authors:** Anna S. Fefilova, Iuliia A. Antifeeva, Anastasia A. Gavrilova, Konstantin K. Turoverov, Irina M. Kuznetsova, Alexander V. Fonin

**Affiliations:** Laboratory of Structural Dynamics, Stability and Folding of Proteins, Institute of Cytology of RAS, 194064 St. Petersburg, Russia

**Keywords:** membrane-less organelles, intrinsically disordered proteins, liquid-liquid phase separation, stress

## Abstract

The discovery of intrinsically disordered proteins (IDPs) that do not have an ordered structure and nevertheless perform essential functions has opened a new era in the understanding of cellular compartmentalization. It threw the bridge from the mostly mechanistic model of the organization of the living matter to the idea of highly dynamic and functional “soft matter”. This paradigm is based on the notion of the major role of liquid-liquid phase separation (LLPS) of biopolymers in the spatial-temporal organization of intracellular space. The LLPS leads to the formation of self-assembled membrane-less organelles (MLOs). MLOs are multicomponent and multifunctional biological condensates, highly dynamic in structure and composition, that allow them to fine-tune the regulation of various intracellular processes. IDPs play a central role in the assembly and functioning of MLOs. The LLPS importance for the regulation of chemical reactions inside the cell is clearly illustrated by the reorganization of the intracellular space during stress response. As a reaction to various types of stresses, stress-induced MLOs appear in the cell, enabling the preservation of the genetic and protein material during unfavourable conditions. In addition, stress causes structural, functional, and compositional changes in the MLOs permanently present inside the cells. In this review, we describe the assembly of stress-induced MLOs and the stress-induced modification of existing MLOs in eukaryotes, yeasts, and prokaryotes in response to various stress factors.

## 1. Introduction

Any organism and, accordingly, its cells are constantly subject to environmental changes that are often stressful. In fact, it is hard to imagine real life conditions lacking occasional stressful impact. Constant temperature, pressure, humidity, etc., is a privilege of the laboratory environment. Throughout its existence each cell and the whole organism must constantly overcome different negative conditions. A failure to adjust to external pressure by the cellular systems leads to various diseases and pathological states at the organismal level.

Cellular stress may be triggered by both physical and biological factors, such as changes in pH, temperature, osmotic pressure, UV radiation, cell cycle disorders, changes in the metabolites and nutrients availability, DNA damage, cellular aging, and various diseases [1]. It should be noted that these factors are interrelated. Thus, a change in the cytoplasmic pH in eukaryotic cells can be caused by osmotic and thermal shock, as well as by the processes of aging and carcinogenesis [2].

The adaptive response of a cell to stress is the activation of various signaling pathways that are specifically determined by the type and severity of injury [1]. For eukaryotes, the most typical pathways are the heat shock response (HSR), unfolded protein responses of the mitochondria (UPR^MT^), the unfolded protein responses of the endoplasmic reticulum (UPR^EM^), and integrated “general” stress response, which is activated by a wide range of physiological conditions, such as amino acid deficiency, viral infection, and endoplasmic reticulum stress [3]. For many serious diseases, such as cancer, viral infection, and neurodegeneration, the association between the disease onset and the disruption of cellular stress response has been proven [4]. For example, inactivation of p53 in cancer, "hijacking" of cellular stress responses by viruses to increase the rate of replication by increasing the number of chaperones, and mutation of key signal transducers such as ATF6 in UPR in neurodegenerative diseases [4].

Regardless of the type of cellular response, stress conditions cause global arrest of the gene expression and protein synthesis, inhibition of most of the "normal" signaling pathways, activation of autophagy, accumulation of a large number of unfolded, partially unfolded, misfolded proteins and RNA that have not been translated [4]. Revolutionary changes in the ideas about the organization of the intracellular space that occurred in the mid-2010s made it possible to form a unified view on the molecular mechanisms underlying cell physiology [5]. First, it became obvious that adaptive, fast, and reversible reprogramming of regulatory pathways in response to a stimulus is achieved with the help of the formation/disassembly of liquid-droplet compartments and, secondly, the concentration of proteins via phase separation is necessary for this mechanism [6]. Intrinsically disordered proteins play a central role in these processes. The structure of disordered proteins presents an ensemble of different conformers, which simultaneously co-exist in solution, and dynamically transits between different conformational states separated by low energy barriers. Due to conformational heterogeneity and the presence of low complexity domains in IDPs sequences, these proteins are capable of spontaneous phase separation in highly concentrated solutions and are the main drivers of MLOs formation [7,8]. Additionally, the promiscuity and plasticity of binding allow IDPs to interact with multiple partners in networks of protein interactions and provide important functional advantages in molecular recognition through transient protein–protein interactions [9]. Short interaction-prone segments within the IDP, called molecular recognition tags, are potential binding sites that can undergo a disorder-to-order transition when binding to their partners [9]. The polyvalence of IDP depends on the cooperation of many separate, weak, non-covalent interactions that combine to give a highly specific end state [10].

**Figure 1:**
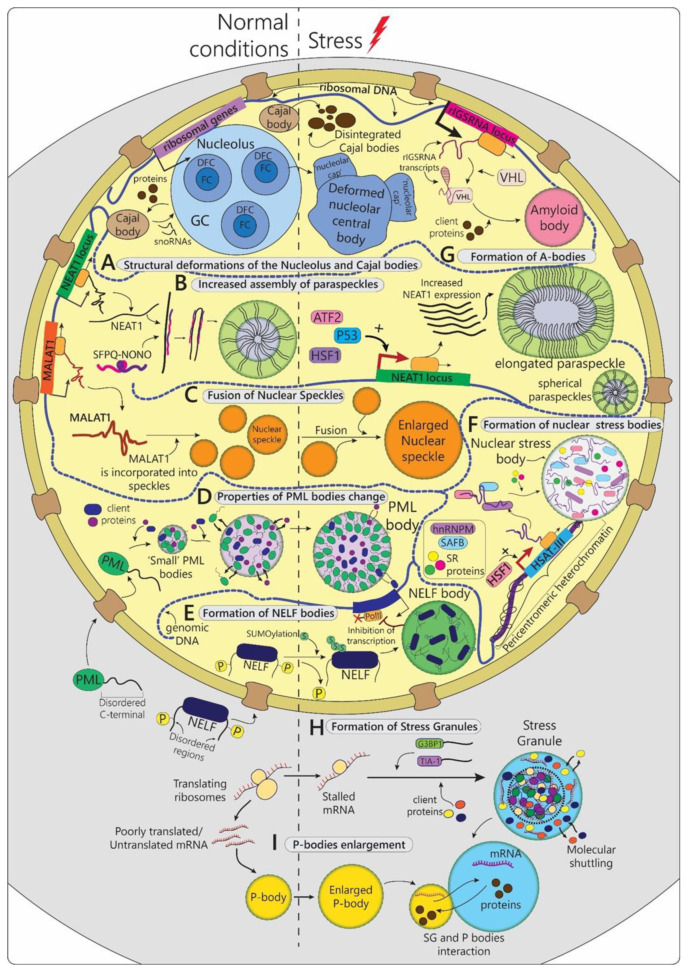
Illustration of biomolecular compartments formed or rearranged in response to stress in eukaryotic cells (**A**–**D**) Nuclear MLOs that undergo structural and functional changes in response to stress. (**A**) Nucleolus and Cajal bodies are structurally deformed in response to stressful stimuli. Under some types of stress, nucleolus loses its tripartite structure while nucleolar central bodies surrounded by nucleolar caps appear. Cajal bodies are reduced in size and/or disintegrate. (**B**) Under normal conditions, paraspeckles assemble due to NEAT1 lncRNA and SFPQ-NONO heterodimer interactions. In response to stress paraspeckles increase in size and numbers as a result of enhanced NEAT1 transcription. NEAT1 transcription is activated by various stress-sensitive transcription factors, such as HSF1, p53, ATF2. Burst in the amount of NEAT1 transcripts leads to the formation of more spherical paraspeckles as well as the assembly of so-called elongated paraspeckles that were suggested to be a result of block copolymer micellization. (**C**) Nuclear speckles, that incorporate MALAT1 lncRNA, during stress increase in size but decrease in number, which is suggested to be a result of their fusion. (**D**) PML bodies that are formed by multiple isoforms of PML protein, upon stress significantly change properties. For example, under H_2_O_2_-induced oxidative stress PML bodies increase in size while the mobility of their components reduces. (**E**–**G**) Stress-induced nuclear MLOs. (**E**) NELF bodies form anew in response to stress after removal of inhibitory phosphorylation tag from the NELF protein and its subsequent SUMOylation. These modifications allow NELF to phase separate and form NELF bodies at the active transcription sites. NELF bodies inhibit RNA Pol II activity downregulating gene expression. (**F**) Nuclear stress bodies (nSB) form with the onset of stress after HSF1 factor activates transcription of HSatIII lncRNA from pericentromeric heterochromatin regions. HSatIII transcripts interaction with HSF1 and other protein results in condensation and assembly of nSBs. (**G**) A-bodies form in a nucleolus vicinity or within it as a result of rIGSRNA transcription. rIGSRNA is transcribed from the intergenic regions of the ribosomal DNA during stress. Increased local concentration of nascent rIGSRNA sequester VHL and other amyloidogenic proteins that together drive assembly and solidification of A-body. (**H**,**I**) Cytoplasmic MLOs involved in stress regulation. (**H**) Stress granules are stress-induced cytoplasmic MLOs that require accumulation of stalled initiation complexes for assembly. SG proteins, such as G3BP1 and TIA-1, are recruited by the repressed mRNA, a process that promotes their phase separation. Formed stress granules have two organizational layers—the low-dynamic core and highly dynamic external shell. The shell actively exchanges the mRNA and protein content with the surrounding cytoplasm (**I**) P-bodies in unstressed cells sequester poorly translated and repressed mRNAs for degradation. During stress, P-bodies enlarge in size and are able to approach stress granules and perform mutual content exchange via direct interaction.

The transformability and pliability of MLOs, provided by unique properties of IDPs composing them, greatly benefit cellular systems ensuring quick and timely response to life-threatening challenges. A clear illustration of that is fast reorganization of cellular compartmentalization under stress conditions. [6]. Initially, the majority of studies devoted to stress-induced MLOs focused on cytoplasmic compartments, especially stress-granules. However, an increasing number of reports have been published demonstrating a multiple nuclear MLOs sensitive to stress and potentially involved in stress-response mechanisms. Some MLOs are stress-induced and form de novo in response to stress, whereas others exist and function in unstressed cells and during stress-response undergo adaptive structural and functional changes ((Table 1, Figure 1) [6,11]. Stress-induced MLOs have been found across eukarya and bacteria life domains advocating early evolutionary development of this cell survival strategy (Table 1, Table 2 and Table 3). In this review, we attempted to classify and give general description of the MLOs formed anew or reorganized during stress-response in prokaryotic and eukaryotic cells and summarize the available data from a unified point of view.

## 2. Eukaryotes

In eukaryotic cells, phase-separated biopolymers undergo significant structural alterations that affect the regulation of stress-specific signaling pathways (Table 1, Figure 1). Some of the stress-responsive MLOs function in the unstressed cells (nucleolus, Cajal bodies, P-bodies, etc.) and upon stress, they undergo significant alterations of properties and potentially performed roles, while other condensates are only present in cells that experience stress or recover from it (cytoplasmic stress granules, A-bodies, etc.) and, thus, are specifically required to combat stress (Figure 1). Moreover, inhibition/activation of the corresponding stress receptors is often accompanied by the formation of biomolecular condensates on the surface of cell organelles [12]. Additionally, reprograming of gene expression programs in stressed cells is associated with the formation of super-enhancers, complexes necessary for activating the transcription of the corresponding genes, as a result of phase separation [13]. Therefore, phase separation is widely used by eukaryotic cells to promote survival during unfavorable conditions.

### 2.1. Nuclear MLOs

#### 2.1.1. Nucleolus

**Figure 2:**
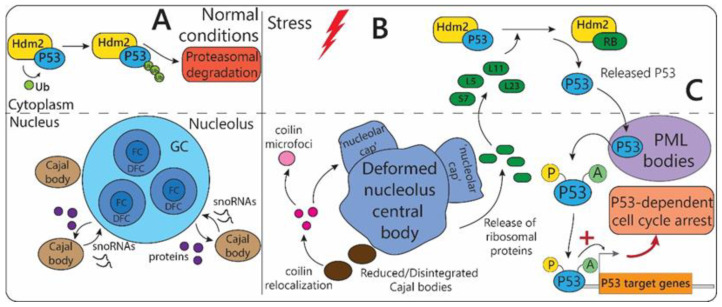
Functional and structural changes in nucleolus and Cajal bodies in response to stress. (**A**) Under normal environmental conditions, nucleolus and CBs are formed in the nucleus via mechanisms of phase separation. Nucleolus is a multiphase compartment composed of three internal layers: fibrillar center (FC), dense fibrillar component (DFC), and granular component (GC). Close proximity allows for content (snoRNAs and proteins) exchange between CBs and nucleolus. P53 is inhibited by direct binding of Hdm2 E3 ubiquitin ligase, ubiquitination by it and degradation by proteasome, and also by export to the cytoplasm. (**B**) The onset of stress results in structural deformations of the nucleolus and CBs. Coilin relocates from disintegrated CBs into ‘nucleolar caps’ or special microfoci. Released from the nucleolus ribosomal proteins displace p53 from p53-Hdm2 tandem via mechanism of competitive binding. (**C**) Activation of p53 in response to stress. Released p53 is relocated to the nucleus, first to PML bodies, where post-translational modification necessary for p53 activation takes place. Then, active p53 binds to promoters of its target genes, initiating cell cycle arrest.

The nucleolus is a dynamic subnuclear structure which has primarily been known for its role in ribosome biosynthesis but has recently gained attention for its novel role in sensing and coordinating cellular stress response. The numerous protein, DNA, and RNA components are spatially organized in three distinct sub-nucleolar compartments, corresponding to the steps of the ribosome biogenesis (Figure 2A): (1) pre-rRNA transcription from rDNA occurs in the fibrillar center (FC) or at the border between the FC and dense fibrillar component (DFC), surrounding the FC; (2) rRNA processing occurs in DFC; (3) pre-ribosome subunit assembly takes place within the granular component (GC), encapsulating FC and DFC. FCs are enriched in components of the RNA Pol I machinery, such as UBF. The DFC component is enriched in pre-rRNA processing factors, such as snoRNPs, fibrillarin, and Nop58. GC is an accumulation of dense particles with a mean diameter of 10–20 nm, which correspond to the most mature precursors of ribosome subunits. The GC is enriched with the protein nucleophosmin (NPM1) [14], which is also involved in ribosome biogenesis [15,16].

Nucleolus morphology, structural integrity, and composition are heavily affected by different stressful stimuli (Table 1, Figure 1A). Two types of nucleolus stress-induced structural deformations have been described: segregation and fragmentation [16]. Nucleoli segregate in response to DNA damage (e.g., UV light [17]) or inhibition of rRNA transcription (e.g., RNA Pol I or topoisomerase II impairment [18]). This process involves condensation with subsequent separation of the FC and GC, accompanied by the formation of ‘nucleolar caps’ around the so-called central body (nucleolus deformed residue) (Figure 1A) [16,19]. On the other hand, inhibition of RNA Pol II or protein kinases leads to the unravelling of the FC, the process called nucleolar fragmentation [20,21].

One of the most prominent mechanisms of nucleolus-dependent regulation of stress response is associated with stabilization and activation of “genome guardian” tumor suppressor p53 (Figure 2B) [22]. Under normal conditions, the p53 function is blocked by inhibitory binding of E3 ubiquitin ligase Hdm2 (also called Mdm2 in mice), which interacts with the p53 transcription activation domain, preventing it from inducing its target genes. Moreover, Hdm2 shuttles p53 from the nucleus to the cytoplasm, a process facilitated by the export of ribosomal subunits [23], where ubiquitinylated p53 can be degraded by the proteasome. In either case of nucleolar segregation or fragmentation triggered by stress, aberrant expression and re-localization of many ribosomal proteins (RBs) are observed. These alterations in ribosome biogenesis initialize p53-dependent cell cycle arrest via several different mechanisms: (1) p53 release from the complex with Hdm2 (Figure 2B); (2) enhancement of the p53 translational profile; and (3) inhibition of co-ribosomal export of p53-Hdm2. The first mechanism is underliedby the competition between p53 and released from the nucleolus RBs for Hdm2 binding, leading to the recession of p53 proteasomal degradation. For example, under ribosomal stress, liberated ribosomal proteins (such as L5, L11, L23, and S7) directly interact with Hdm2 blocking its association with p53 (Figure 2B) [16,24]. Then, the elevation of active p53 levels under stress conditions is also facilitated by its increased translation. For instance, under genotoxic stress, the released L26 from the 60S ribosomal subunit ribosomal protein binds to the 5’ untranslated region of p53 mRNA and upregulates its translation [25]. Under normal conditions, the association of L26 with p53 mRNA is additionally repressed by Mdm2-induced polyubiquitylation and proteasomal degradation of L26. However, under genotoxic stress, this process is inhibited [25]. Finally, the last described pathway involves inhibition of p53/Hdm2 co-export with ribosomal subunits from the nucleolus to the cytoplasm where p53 proteasomal degradation occurs [23].

It is known that many different viruses target proteins to the nucleolus and recruit nucleolar proteins to facilitate virus replication. It obviously affects the morphology and composition of the nucleolus. For example, the coronavirus infection increases nucleolar size and, in particular, the enlargement of FC, as well as alternates the nucleolar proteome (e.g., localization of nucleocapsid (N) protein of coronavirus to the DFC of nucleolus, an increase in the amount of nucleolin within nucleolus) [26]. Viral infections may also induce the nucleolar accumulation of chaperones such as Hsp70. The Hsc70s (heat shock cognate proteins 70) are located to the nucleolus during the recovery period after stress [27]. It has been shown that under cellular starvation in the serum-free medium, the level of nucleophosmin in the nucleoli was diminished while its amount in the nucleoplasm increased. When the normal serum content has been restored, the nucleophosmin relocated back to the nucleolus [28]. Additionally, a wide range of anticancer agents induced the nucleoplasm translocation of nucleophosmin [15].

#### 2.1.2. Cajal Bodies

Cajal bodies (CBs) are nuclear MLOs that have been functionally linked to the nucleolus. CBs are often observed in a close spatial proximity to the nucleolus (and in some cases even within it) [29]. They share a certain degree of compositional overlap (for example, proteins fibrillarin, nucleolin, Nopp140, NAP57); moreover, the constant flux of proteins and various RNA species between these two nuclear entities has been revealed (Figure 1A and Figure 2A) [30]. CBs are involved in the maturation of small nucleolar RNA (snoRNAs) which are necessary for rRNA post-transcriptional modifications. In this way, CBs facilitate the nucleolus in rRNA biogenesis. Given that functional and spatial interconnection of nucleolus and CBs and the nucleolar role in the stress response, it should not be a surprise that CBs are also responsive to stress [16]. Besides snoRNAs, CBs are also centers for small nuclear RNA (snRNA) and histone mRNA processing. CBs assemble at snRNAs transcriptional loci and sometimes at sites of active histone mRNA transcription [29]. Additionally, a distinct type of small non-coding RNAs, called scaRNAs (small Cajal body-specific RNAs), is specifically localized to CBs. scaRNAs guide RNA modifications on snRNAs [29].

CBs are conserved MLOs found in plant and animal cells. Additionally, structures compositionally and functionally similar to CBs have been reported in other organisms. The major CBs scaffold protein coilin is widely used as a molecular marker of CBs. However, in some organisms (e.g., Drosophila, C. elegans, yeast), coilin or its obvious homologues are absent which impedes the CBs homologues identification. In budding yeast, the analogue of CBs named “nucleolar body” is found within the nucleolus. These bodies are enriched with the same components as mammalian CBs such as precursor forms of U3 snoRNAs and TGS-1 (conserved methyltransferase catalyzing the formation of the 5’ terminal tri-methyl-CAP structure in sno- and snRNAs) [31]. 

Coilin-deficient animals (flies, mice) and plants (Arabidopsis) lack CBs; however, they still remain viable [32,33,34]. On the other hand, coilin gene disruption (and therefore CBs loss) is semi-lethal for zebrafish and murine embryos (especially late in the gestation period when embryos rapidly grow) [32]. Additionally, coilin knockout mice display reduced litter size and litter number, compared to wildtype controls, and mutant males have smaller testes, which could reduce or delay sperm production and mutant females might produce fewer mature oocytes [32]. Embryonic fibroblasts derived from these animals lack typical CBs but contain residual bodies containing a subset of typical CB components [35]. For zebrafish embryos, functional CBs are absolutely required for completion of the developmental process and concomitant cell survival [36]. Depletion of coilin in zebrafish embryos leads to splicing defects that could be partially restored by injection of fully assembled snRNPs [36]. Thus, according to the collected data, CBs are not essential for the developed organism under normal conditions. At the same time, these cellular structures are highly conserved and withstood a great evolutionary pressure and, therefore, bear a significant natural selection benefit. This allows us to suggest that CBs’ key role may lie in the maintenance of the cellular homeostasis in abnormal or quickly changing conditions, as well as for highly specific parts of the life cycle, such as embryogenesis. For instance, the suppression of coilin gene expression can confer salt tolerance on *N. benthamiana* plants, confirming the role of CBs in the plant cells response to osmotic shock [37].

Typically CBs disintegrate in response to various types of stress with its core proteins being relocated (e.g., coilin [38,39]) or undergo proteasomal degradation (e.g., FLASH protein [40]) (Table 1, Figure 1A and Figure 2B). It has been shown that cellular starvation decreases the number and size of CBs [41]. The UV-C irradiation, osmotic stress, and heat shock reversibly disrupt CBs, with the formation of the coilin-containing nucleoplasmic microfoci (Figure 2B) [38,39]. The chilling stress of soybean root meristem cells reduces the number of CBs with the subsequent recovery of their amount after the stress. However, this reduction may be caused by the hindering of CBs formation or by their fusion [42]. The CBs disassembly may be caused by the alteration of intermolecular interactions associated with the stress-induced posttranslational CB proteins modifications (e.g., SUMOylation of CB proteins upon stress [43]) and/or by the degradation of CB components via the proteasomal pathway. Thus, the involvement of proteasome activator subunit PA28g in the UV-C-induced coilin nuclear redistribution was clearly demonstrated [38]. It has also been shown that coilin is not degraded during stress, as its cellular levels remain constant, but rather it changes its localization. Thus, the inhibition of RNA polymerase II transcription by 5,6-dichloro-1-b-D-ribobenzimidazole causes the transition of coilin into the cap-like structures associated with the nucleolus (Figure 2B) [16]. 

It has been shown that different viral infections lead to diverse CBs responses. For example, HSV-1 infection induces the relocation of some CBs proteins (coilin, SMN, and fibrillarin) to the damaged centrosomes [44]. Adenoviruses induce the redistribution of the coilin and some other CB components in the periphery of viral replication centers to participate in the processing of virial transcripts [45]. In plants, groundnut rosette virus (GRV) induces the fusion of the transformed CBs containing viral ORF3 protein with the nucleolus [46]. However, the data regarding the functional importance of these structural changes have been rather contradictory. For example, it was reported that knockdown of coilin in Nicotiana plants may increase the accumulation of the barley stripe mosaic virus and tomato golden mosaic virus promoting the virus spread. On the other hand, the same study using the same knockdown system reported a decline in virus accumulation in the case of the turnip vein clearing virus and the potato virus Y, also linked to downregulation of symptoms progression [19,37].

Overall, the available data suggest that CBs are highly sensitive to various types of stress. However, the question remains whether the observed structural alterations are a consequence of cell response to stress or a part of its regulation and if the latter, then the exact mechanisms are awaiting clarification.

#### 2.1.3. Paraspeckles

**Figure 3:**
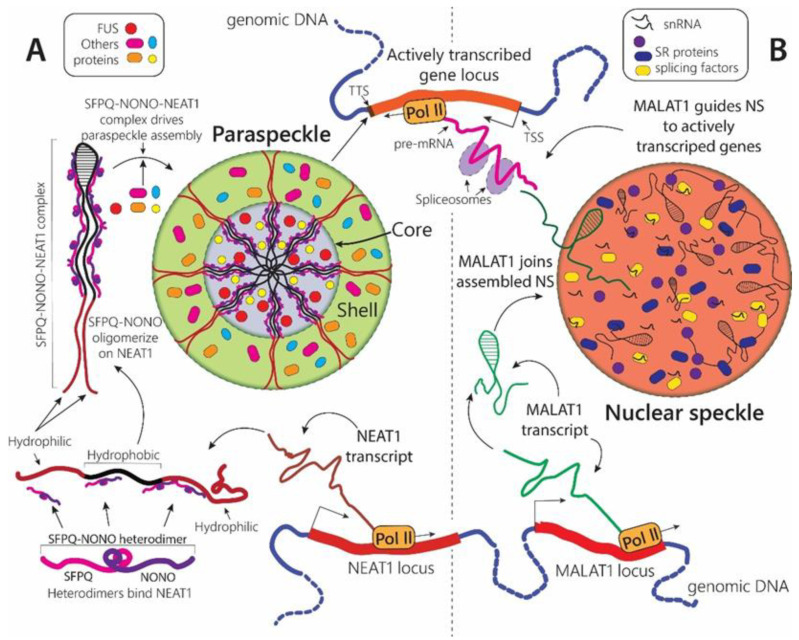
Assembly and cooperation of nuclear speckles and paraspeckles. (**A**) Paraspeckles assemble at sites of NEAT1 lncRNA expression. Nascent NEAT1 transcripts sequester SFPQ-NONO heterodimers that oligomerize on the synthesized NEAT1, stabilizing it and forming SFPQ-NONO-NEAT1 complexes. These tripartite complexes assemble into the condensate that attracts multiple client proteins, forming mature paraspeckle. The hydrophobic and hydrophilic regions of NEAT1 fluctuate towards center and edges of paraspeckle, respectively, forming the core and the shell. (**A**) MALAT1 lncRNA is incorporated into nuclear speckles but is not required for their formation. NS contains various splicing factors, and it was found that MALAT1 accumulates at the sites of active transcription, potentially guiding NS to the spliceosomes. (**A**,**B**) NEAT1 and MALAT1 are expressed from the adjacent genomic sites. NS and paraspeckles colocalize at the actively transcribed gene loci. Paraspeckles are more enriched at transcriptional start sites (TSS) and transcriptional termination sites (TTS). NS primarily localized across gene bodies.

Another example of MLOs that respond to stress with structural and functional changes is paraspeckles (Table 1, Figure 1B and Figure 3A). Paraspeckles are nuclear condensates which assembly is driven by the long noncoding RNA NEAT1 (Nuclear Paraspeckle Assembly Transcript 1). NEAT1 is a single-exon transcript that is alternatively spliced in human cells to produce short 3.7-kb (NEAT1_1) and long 22.7-kb (NEAT1_2) isoforms. Long NEAT1_2 is essential for paraspeckle formation. Its knockdown with antisense oligonucleotides resulted in a complete disintegration of paraspeckles in both human and murine cells [47]. The paraspeckles most probably assemble co-transcriptionally at the nascent NEAT1 RNA, however, they may migrate throughout the nucleoplasm upon maturation. The process of paraspeckle formation starts with the expression of NEAT1 transcripts followed by binding of the members of DBHS (Drosophila Behavior Human Splicing) family—proteins SFPQ and NONO—which together form SFPQ-NONO functionally active heterodimers (Figure 1B and Figure 3A). The initial binding of SFPQ and NONO to NEAT1 is essential for NEAT1 stability. Additionally, SFPQ-NONO represent paraspeckle structural scaffold themselves as RNA-protein interaction leads to oligomerization of SFPQ-NONO heterodimers into longer chains of polymers along NEAT1_2 transcripts increasing the system multivalency (Figure 3A). The knockdown of either SFPQ or NONO completely oblates paraspeckle formation [48]. At the final step of assembly, the SFPQ-NONO-NEAT1 system attracts the additional proteins such as FUS and the phase separates, forming the mature paraspeckle [49]. The paraspeckles are composed of a core part containing the middle hydrophobic part of NEAT1_2 transcripts and the shell part containing the 5′ and 3′ hydrophilic ends of NEAT1_2 [49,50] (Figure 1B and Figure 3A). The core and the shell also have different protein compositions. Interestingly, paraspeckles can become elongated, forming cylindrical shapes over time (Figure 1B and Figure 3A).

The paraspeckle assembly has been tightly linked to cellular adaptation to changing external conditions. Working as a storage hub for RNAs and proteins involved in the transcription regulation and pre-mRNA processing, paraspeckles modulate various cellular pathways, such as circadian cycling and response to various stressors (mitochondrial stress, hypoxia, heat shock, viral infection, etc.). During normal conditions, when cells are unstressed, paraspeckles are still ubiquitously observed in cellulo but not in vivo. In mice raised in stable laboratory conditions, paraspeckles are rarely found within tissues, and usually appear in terminally differentiated cells such as at the tips of crypts in the large intestine or corpus luteum [48,51]. It has been shown that NEAT1 knockout (KO) mice, which lack paraspeckles, are viable and fertile, however, nearly half of the naturally mated female mice stochastically failed to become pregnant probably due to the dramatic decrease in serum progesterone level due to corpus luteum impairment in the KO animals [51]. In cell culture, paraspeckles were reported in all the cell types except embryonic cells; however, their differentiation was shown to be accompanied by the paraspeckles formation [48]. Altogether these data indicate that paraspeckles, while not vital MLOs, aid cells in adjusting to specific, not yet clearly identified, changes in environmental conditions as well as in the internal cellular state.

Most cells can reversibly multiply the number of paraspeckles upon different types of stress (Figure 1B). The increase in the paraspeckles level has been shown under hypoxia conditions [52], temperature elevation [53], sulforaphane treatment [53], as well as for softening of the cellular substrate [54]. The number of nuclear paraspeckles correlates with the amount of the expressed NEAT1_2, while corresponding protein levels remain unchanged. Therefore, the stress-dependent accumulation of paraspeckles is triggered by enhanced transcription of NEAT1, activated by various stress-responsive transcription factors, such as HIF-2α during hypoxia [52], HSF1 during heat shock [53], p53 in replication stress [55], or ATF2 during mitochondrial stress [56], each of which binds to the corresponding element located in the NEAT1 promoter (Figure 1B).

It has also been shown that viral infections predominantly increase the paraspeckles number. The elevated amount of paraspeckles enhances the sequestration of the SFPQ protein which is a suppressor of several anti-viral genes (e.g., RIG-I and IL-8). Such sequestration causes the de-repression of the respective genes with the following production of the gene products. This mechanism has been observed for Hepatitis D, Influenza, polyI:C infection, and Hantavirus [57,58,59]. Moreover, paraspeckles are involved in the nuclear retention of the viral mRNA, for example, REV-dependent HIV-1 transcripts [60]. Paraspeckles also play an essential role in the antibacterial immune response. For example, upregulation of NEAT1 is observed in response to salmonella infection [61]. 

The paraspeckles are observed in two distinct shapes: spherical shape, typical for other MLOs, and unusual elongated shape (Figure 1B) [49]. Some stress events trigger the formation of spherical paraspeckles (temperature [52], hypoxia [53]), while others the formation of elongated paraspeckles. Thus, mitochondrial stress caused by depletion of mitochondrial proteins leads to the generation of elongated paraspeckles [56]. The shift from sphere to cylinder-like shape has been associated with alterations in post-transcriptional processing of the NEAT1-favoring production of long NEAT1_2 over short NEAT1_1 [49,56]. This is in accordance with the suggested block copolymer micellization model of paraspeckles elongation in which cylindrical micelles depend on the NEAT1_2 level and are stabilized above its certain threshold [50] (Figure 1B). The dynamic of the micellization process is distinct from that of the liquid-liquid phase separation and was suggested to facilitate the regulation of paraspeckle size [50]. Thus, paraspeckles are the only currently known MLOs that may assemble not as a result of the LLPS process alone. However, if it is indeed the case, then it is reasonable to expect a discovery of analogical assembly mechanisms for other biological condensates. 

#### 2.1.4. Nuclear Speckles

Nuclear speckles (NS) are nuclear MLOs involved in splicing regulation. NS are also sometimes called ‘interchromatin granule clusters’ as they are located in the interchromatin regions of the nucleoplasm of mammalian cells. NSs contain pre-mRNA splicing factors, including snRNPs and SR proteins [62]. Additionally, long non-coding RNA MALAT1, a single-exon transcript over 7 kb in length, is enriched in NS through its specific interactions with NS-retained proteins (Table 1, Figure 1C and Figure 3B). MALAT1 was found to regulate the SR splicing factors distribution to NS via direct interaction and modulation of their phosphorylation state [63]. SR proteins cycle between phosphorylated and dephosphorylated states, which is essential for pre-mRNA processing. MALAT1 depletion results in both dephosphorylation of SR proteins and differential changes in alternative splicing events in several mRNAs, mostly exon inclusions [63]. However, MALAT1 is dispensable for NS formation or cellular viability and MALAT1-deficient mice did not demonstrate abnormalities in alternative splicing patterns [64].

It was demonstrated that MALAT1 localizes to actively expressed genomic loci, most likely via its proteins partners targeting long non-coding RNA to newly synthesized pre-mRNA transcript (Figure 3B) [65,66]. Additionally, with the help of various genome mapping methods, it was uncovered that NS are associated with chromosome regions characterized by high levels of active RNA polymerase II transcription [67,68]. These discoveries have led to the suggestion that MALAT1 acts as a molecular leash delivering splicing machinery contained in the NS at the sites of active gene transcription [66,69] (Figure 3B). Moreover, it has been experimentally shown that association of Hsp70 genes and four genes flanking the Hsp70 locus with nuclear speckles causes a several-fold boost in expression of these genes following heat shock [68]. Authors suggested that this NS-dependent upregulation is a result of the decreased exosomal degradation of the nascent transcript combined with increased transcriptional rate [68]. Based on these results, a so-called “gene expression amplification” model was proposed. According to this model, nuclear speckles act as gene expression hubs capable of increasing the net production of transcripts of genes positioned in the NS vicinity [68].

Interestingly, the core RNA of paraspeckles NEAT1 is positioned in the genomic environment of MALAT1 and two RNAs are transcribed from the adjacent regions in the genome [69] (Figure 1B,C and Figure 3A,B). Despite that, these RNAs partition to different MLOs and never colocalize to the same condensate. On the other hand, the nuclear speckles and paraspeckles has been found to localize together at hundreds of active gene loci, however, primarily bound to distinct parts of the genes: NEAT1 was found at transcriptional start sites (TSS) and transcriptional termination sites (TTS), whereas MALAT1 primarily localized across gene bodies [69] (Figure 3A,B). This might indicate a cooperation between these two biological condensates in the upregulation of gene expression. It is not yet clear if this mutually functional complementarity is maintained during the stress condition.

NS accumulates various splicing factors as well as components of the splicing machinery. The alternative mRNA splicing is significantly impacted by stressful stimuli via changes in localization, interactions, expression, and chemical modifications of splicing factors and spliceosome components [70]. Additionally, changes in alternative splicing patterns are used by cells to regulate gene expression in order to combat stress [70,71]. For a significant part of the transcriptome, splicing is downregulated in response to heat shock with the exception to genes involved in stress response [71]. NS are inevitably involved in these regulatory pathways; however, specific mechanistic details remain unknown.

Similar morphological changes have been observed in NS across various stress conditions. Typically, enlargement and rounding of NS condensates accompanied by the reduction in their total number is reported (Figure 1C). This has been shown for transcription arrest caused by heat shock (45 °C for 15 min) [72], treatment with transcription inhibitors, such as actinomycin D [73,74], genotoxic stress induced by Etoposide [75], heavy metal stress [74], and osmotic stress [76]. This aberrant morphology was attributed to two processes: 1) proteins migrating back to NS for storage upon stress [75]; 2) NS particles fusion [74] (Figure 1C). Moreover, increased NS mobility, characterized by long-range directional migration across interchromatin space was demonstrated for several different types of stress [74]. Interestingly, this motion terminates with condensates coalescence, suggesting that NS mergence is not a stochastic, but rather a controlled process [74].

#### 2.1.5. PML-Bodies

PML bodies are nuclear polyfunctional compartments that are involved in the regulation of transcription, stress response, differentiation, and transition of cells to the senescent state and are present in cells under normal conditions [77]. The major protein of these compartments is the promyelocytic leukemia (PML) protein (Table 1, Figure 1D). The main components of PML bodies in human cells are the six nuclear isoforms of the protein of promyelocytic leukemia (PML) formed via alternative splicing, therefore, they differ in size and amino acid sequence of their C-terminal domains [78,79,80].

Analysis of morphology and dynamics of PML bodies showed the existence of at least two populations of PML bodies in U2OS and HeLa cells with a diameter of about 0.6 µm and 1.2 µm. In the population of “small” PML bodies, all bodies are spherical and all PML isoforms dynamically exchange with nucleoplasm. It has been suggested that such bodies act as liquid “seeds” of functionally active PML bodies, forming due to weak intermolecular interactions and providing the necessary concentration of PML isoforms for the formation of intermolecular disulfide bonds between PML monomers (Figure 1D). The “large” mature bodies have a toroidal morphology and scaffold with low mobility formed predominantly by PML-V and PML-VI [81,82].

PML bodies are one of the key regulators of the p53-dependent stress response (Figure 2C) [83]. In response to stress, p53 undergoes a number of post-translational modifications necessary for the activation of this protein and the subsequent induction of the expression of cyclin-dependent kinase inhibitor genes, which, in turn, contribute to the inhibition of proliferative gene expression and cell cycle arrest [84]. Nuclear PML bodies are one of the main platforms that provide the post-translational modifications of this protein necessary for the activation of the p53-dependent signaling pathway (Figure 2C) [77]. PML bodies promote activation of p53 target genes which are oxidative stress-induced, for example, Trp53inp1 or Sesn2 are part of the p53 anti-oxidant response [83]. According to recent data, the PML-IV isoform makes a decisive contribution to p53 activation, forming PML-IV-CBP-p53 complexes in PML bodies [85]. Under hypoxic conditions, PML bodies suppress the AKT-mTOR signaling pathway by inhibiting PP2 phosphatase within these organelles [86]. PML bodies also promote activation of the DNA damage response via the ATM/ATR-p53-p21 pathway [78].

According to the previously existing model of PML bodies formation [87,88], oxidative stress should induce solidification of PML bodies due to the disulfide-mediated multimerization of PML monomers and enhancement of intermolecular electrostatic interactions by K487 deacetylation and K490 SUMOylation [89,90]. However, using the FRAP method to characterize liquid properties of condensates, it has been shown for PML-/- HeLa cells as well as for wild-type cells, that oxidative stress induced by H_2_O_2_ alters the dynamicity of the main proteins of canonical PML bodies, as well as the PML bodies associated with alternative telomere lengthening (APBs) (complete immobilization of PML-V and decrease mobility of PML-I and PML-II between nucleoplasm and these organelles) while its localization and morphology are still practically unchanged [81,82]. Peroxide treatment of U2OS cells causes a slight increase in the size of APBs. The exchange rate of PML-III, PML-IV, and PML-VI between PML bodies and nucleoplasm remained unchanged upon oxidative stress. At the same time, hydrogen peroxide treatment completely immobilized the PML-V isoform within PML bodies and reduced the diffusion of PML-I and PML-II isoforms. The arrested PML-V diffusion may be caused by or promote the disulfide bonds formation between these isoforms, which is forced by a strong tendency for its α-helical motif to form hyper stable oligomers and a low diffusion rate of this isoform under normal condition [81]. The dynamic of the C-terminal domain of PML-II and PML-V as well as their mutants with K490R substitution, disrupting the PML SUMOylation, in normal and oxidative stress conditions caused by H_2_O_2_ treatment has also been studied. A slight decrease in the exchange rate and a decrease in the proportion of the mobile fraction of the wild-type PML-II C-terminal domain have been observed. For the mutant form of the PML-II C-terminal domain with the K490R substitution, a slight increase in the exchange rate has been revealed. Additionally, oxidative stress caused a significant decrease in the diffusion rate of the C-terminal domain of PML-V and its mutant form with the K490R substitution between the bodies and the nucleoplasm. The dynamics of the exchange of the mutant form of the C-terminal domain of PMLV, K490R, under the conditions of the acute oxidative stress, slows down significantly more than that of the wild-type domain [82]. The induction of oxidative stress by As_2_O_3_ resulted in degradation of most of the PML isoforms, leaving the SUMO at the core of the nuclear bodies. PML-I, PML-II, and PML-VI isoforms dissociated to cytoplasm upon arsenic treatment [79]. The exposure of cells to other types of stress such as heat stress, heavy metal addition, and expression of adenovirus E1A demonstrated the decrease in the number and size of PML bodies and the formation of smaller PML-containing structures called ‘microstructures’. Such microstructures are formed from parental PML bodies as a result of fission or budding from its surface. They are mobile and able to fuse with each other as they move through the nucleoplasm. The over-expression of SUMO-1 prevents the formation of microstructures [27].

During nuclear dissociation during mitosis, PML bodies are not disassembled, but are transformed into the so-called mitotic accumulation of PML proteins (MAPPs), which can be visualized using confocal fluorescence microscopy [91]. In the early G1 phase of the cell cycle, MAPPs, in turn, are transformed into the so-called cytoplasmic assemblies of PML and nucleoporins (CyPN) [92]. CyPNs are large gel-like structures prepared for nuclear import containing KPBN1 importin and at least 20 FG-porins are involved in the formation of a selective barrier inside nuclear pores. Like MAPPs, these structures can be easily visualized using confocal fluorescence microscopy. During the PML translocation into the core, CyPN is disassembled.

#### 2.1.6. NELF-Bodies

Unlike MLOs discussed previously, that can be observed in the nucleus of the unstressed cells, there is also a group of biological condensates only present in cells that experience stress or recover from it. Recently, a novel stress-induced condensate formed by a negative elongation factor (NELF) has been described (Table 1, Figure 1E) [93]. In order to successfully resist stress, cells need to quickly reprogram a multitude of regulatory pathways and shut down processes that are not essential for immediate survival. So, one of the first steps of stress-response is downregulation of transcription and translation. NELF is a negative regulator of transcription that directly inhibits RNA polymerase (Pol) II activity at the elongation step via binding [93,94]. It has been found that NELF is able to undergo LLPS in vitro and upon stress forms nuclear condensates in cellulo, that potentially stabilize its interaction with chromatin and enhance inhibitory potential. NELF protein contains intrinsically disordered regions, so-called “tentacles”, that are essential for its phase separation (Figure 1E) [93]. Under normal conditions, NELF is present in the cell, but its activity is blocked by CDK9-dependent phosphorylation. Upon stress induction, NELF is quickly dephosphorylated and SUMOylated that promotes its condensation and formation of NELF bodies at transcriptional loci of many housekeeping genes [93]. NELF bodies block Pol II enzymatic activity, promoting global downregulation of transcription and aiding cell survival mechanism (Figure 1E) [93].

#### 2.1.7. Nuclear Stress-Bodies 

Nuclear stress bodies (nSBs) form de novo in the cell nucleus in response to stress. nSB assembly starts with the activation of expression of a so-called human highly repetitive satellite 3 long non-coding RNA (HSatIII) [95] (Table 1, Figure 1F). HSatIII contains multiple tandem repeats of nucleotide sequences and is transcribed from the pericentromeric heterochromatin. Its synthesis is triggered by binding of the HSF1 (heat shock factor 1) transcription factor. Increased local concentration of the nascent HSatIII transcripts attracts HSF1 and other proteins providing the platform for nSBs nucleation (Figure 1F) [96]. On average, several 1-2 μm nSBs assembled in one cell, all of them form and remain in a vicinity of HSatIII loci located on several chromosomes. The main protein components of nSBs are the heat shock regulators, HSF1 and HSF2; hnRNP proteins, SAFB and hnRNPM; and other mRNA splicing factors (Figure 1F) [95,96]. nSBs possess properties of a phase-separated condensates. However, they are subject to hardening in the conditions of a prolonged stress that leads to reduced cellular viability [97]. Concentrations of HSF1 and SAFB mark two successive phases in nSBs evolution. HSF1 is predominant during the eruption of the stress response with gradual decline of its levels during a stress recovery period, whereas SAFB is incorporated into nSBs with the delay and peaks after the stress termination [98]. The biological significance of nSBs is not entirely clear. There is evidence that nSBs may be involved in the regulation of mRNA splicing. For example, an increased import of SR proteins into nSBs was detected in response to stress [6]. Additionally, nSBs components positively impact cell survival, thus HSF1 and SAFB knockdowns promoted apoptosis [6]. Overall, further studies are necessary to shed the light onto nSBs biogenesis and the role in the regulation of cell survival during stress.

#### 2.1.8. A-Bodies

Like nuclear stress bodies and NELF bodies, amyloid bodies (A-bodies) assemble transiently in the cell nucleus in response to stress. A-bodies are droplet-like foci containing hundreds of proteins in the amyloidogenic state (Table 1, Figure 1G) [99]. Although solid-like MLOs are often considered pathological, A-bodies formation is reversible and useful for temporal storage of molecules. A-bodies are formed in several stages. At first, the stress (heat, acidosis, etc.) induces synthesis of non-coding RNA molecules called rIGSRNA (ribosomal intergenic spacer RNA). rIGSRNA transcripts are expressed from intron regions of ribosomal DNA consisting of numerous dinucleotide repeats (Figure 1G) [99]. Then, a local increase in the concentration of rIGSRNA molecules, represented by sequences with a low degree of complexity, causes the formation of ‘seeds’ for bimolecular condensates, to which amyloidogenic proteins containing ACM (amyloid-converting motif) are recruited, in particular E3 ubiquitin ligase VHL (Von Hippel–Lindau tumor suppressor). Different types of stress induce transcription from various areas of rIGS and production of distinct rIGSRNA isoforms. In turn, different rIGSRNAs sequester different proteins subsets [100]. Electrostatic interactions between negatively charged low-complexity RNA and disordered positively charged regions of ACM-containing proteins rich in arginine and histidine residues promote condensation [101]. At this stage A-bodies display properties of liquid-like dynamic condensates, such as fusion and content mobility. Then, a high local accumulation of hydrophobic fibrillation propensity domains of ACM creates conditions for the transformation of bimolecular condensates into a gel-like state and then into aggregates of amyloid fibrils [102]. Mature A-bodies completely immobilize stored proteins. The breakdown of A-bodies is initiated after the termination of stress and is carried out in an Hsp70/90-dependent manner [102]. The released proteins do not undergo degradation but change the topology of the polypeptide chain to the native conformation and return to functional state. Thus, the key function attributed to A-bodies is protein storage and the isolation of potentially toxic amyloid fibrils preventing their interaction with the rest of the cellular proteome.

### 2.2. Cytoplasmic MLOs 

#### 2.2.1. Stress-Granules

**Figure 4:**
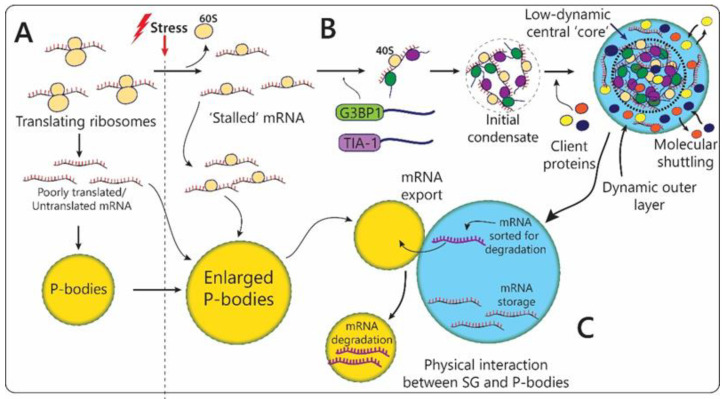
Stress granules and P-bodies interplay during stress response. (**A**) Under normal conditions, SGs are absent from the cytoplasm, mRNA translation is normal, and poorly translated or repressed mRNAs are sequestered into P-bodies. (**B**) Upon stress treatment, translation is inhibited and ‘stalled’ translation initiation complexes are recruited to P-bodies, causing their enlargement, or interact with SG proteins, such as G3BP1 and TIA-1. This interaction leads to phase separation and formation of initial pre-mature SG that then attracts more proteins. Mature SG has a low-dynamic central core and dynamic outer layer. (**C**) Stress conditions promote physical association between SG and P-bodies. It was suggested that within SG, the mRNA molecule undergoes sorting and the mRNA destined for decay are exported directly into P-bodies via temporal fusion between SG and P-bodies.

Stress granules (SG) are cytoplasmic membrane-less organelles that transiently assemble in eukaryotic cells in response to various types of endogenous (for instance, impaired proteostasis, genotoxic stress, etc.) and exogenous stresses: temperature, oxidative stress, UV irradiation, nutrient deprivation, hypoxia, viral infection, and many others [103,104]. They have been found to be the key regulators of cellular stress response, reducing detrimental consequences of stress-induced damage. At least partially, this is achieved via incorporation into SGs translationally stalled mRNAs, RNA-binding proteins, and translation initiation factors and, thus, temporal isolation of these molecules from the rest of the cellular milieu (Table 1, Figure 1H and Figure 4). Translation is one of the most energy-consuming cellular mechanisms, therefore, it is one of the first to be inhibited in response to stress in order to save cellular resources for the stress response. mRNA and translation factors are recruited into SGs upon stress and re-enter normal translation process after normalization of conditions and release from granules, making SGs temporal ‘storage’ capsules for indispensable molecules. Importantly, mRNA coding for stress response factors, such as heat shock proteins mRNA, are not incorporated into stress granules, allowing cells to activate expression of proteins essential for survival [105]. The cytoprotective benefits of such mechanism include global energy savings on mRNA and protein degradation and post-stress resynthesis, as well as hindrance of the toxic aggregation of partially unfolded protein biopolymers in the cytoplasm.

The highly dynamic nature of SGs allows them to quickly modulate cellular translation and proteostasis during unfavorable conditions thus promoting cell survival [106]. SGs have vast therapeutic potential as their deregulation has been linked to progression of multiple neurodegenerative disorders [107], oncogenesis and resistance to treatment of cancer cells [103,108], and viral replication inside the host [109] and other pathologies. 

SGs formation takes several steps, the first of which starts when cellular stress leads to translation arrest via various pathways, including phosphorylation of translation initiation factors eIF2 and eIF4. Abrupted translation causes dissociation of polyribosome accumulation of free mRNA in the cytoplasm, which is then able to interact with RNA-binding motifs of SG scaffold proteins (G3BP1/2, TIA-1, and others), driving their liquid-liquid phase transition and nucleation of initial SG condensates (Figure 1H and Figure 4A,B) [110,111]. Further maturation of SGs relies on higher-order heterotypic interactions between scaffold proteins leading to ‘hardening’ of the central ‘core’ of SG, around which client proteins form a more dynamic layer (Figure 4B) [111]. Functional activity of SGs depends on the composition of the dynamic outer phase.

The liquid droplet properties of SG ensure constant trafficking of molecules between the granule and the surrounding cytoplasm, allowing for a timely response to the onset and termination of stress. Upon restoration of normal conditions, SGs are quickly disassembled, and released mRNA is re-recruited by translation machinery. Decay of SGs is facilitated by chaperones inhibiting the mRNA–SG core proteins interaction [112,113]. Upon termination of stress, SGs can also be degraded via an autophagosomal mechanism [114] while violation of this process can lead to the formation of cytotoxic amyloid fibrils [114,115].

Several neurodegenerative diseases, including amyotrophic lateral sclerosis (ALS), Alzheimer’s disease (AD), and frontotemporal dementia (FTD), are associated with abnormal SG biogenesis. Transformation of SG into toxic aggregates of amyloid fibrils is promoted by incorporation of the disease-associated mutant forms of proteins, such as TIA-1, TIAR, FUS (RNA-binding protein fused in sarcoma), hnRNPA1 (heterogeneous nuclear ribonucleoprotein A1), TDP-43 (transactive response DNA binding protein 43 kDa), and PABP1 (polyadenylate-binding protein 1) [116,117].

RNA is the major component of SG as 78–95% of SG composition are RNA molecules [118]. Despite extensive high throughput analysis of SG transcriptome, the mechanisms that drive the enrichment of certain RNA transcripts into SGs but not the others remain unknown. Previous studies have shown that all cellular mRNAs are represented in SGs to some extent, however, the magnitude of their concentration relative to cytoplasm differs drastically, suggesting that yet unknown factors promote preferential recruitment of certain RNAs to SG [104,118]. One parameter that was found to positively correlate with SG recruitment was the length of the transcript [104]. However, other studies demonstrated that mRNAs of the same length show different levels of SGs incorporation. These data suggest that individual mRNA molecules carry specific information that significantly affects their enrichment into these organelles. This could be attributed to primary nucleotide sequence, secondary structure, modifications of RNA nucleotides and the last especially has been the research focus in recent years. A curious contradiction can be found between two papers published recently [119,120]. Both studies performed a comparative analysis of mRNA partitioning into SG between wildtype and METTL3 methyltransferase knockout mouse embryonic stem cells (mESC). METTL3 is the key writer enzyme of m6A RNA modification, the most common type of chemical modification found in mRNA. One of the reports showed an association between m6A modifications and average mRNA enrichment into SG [119]. However, a report published by Khong et al. found no evidence that METTL3 depletion affects mRNA composition of SGs, leading to the conclusion that m6A edits play little or no role in this process [120]. This contradiction can be explained by a deeper assessment of the properties of the METTL3 knockouts used by the two groups. The study that suggested positive correlation between m6A modifications and mRNA SG enrichment used knockout cells with a complete loss of m6A upon induced deletion of METTL3. On the other hand, the other work was performed on METTL3 knockout mES cell line that only had partial loss (~60%) of m6A levels. Moreover, this knockout cell line has been found to have an activated expression of a shortened partially functional METTL3 isoform [121]. Altogether, these data suggests that m6A chemical markers, and potentially other types of RNA modifications, are important for SG transcriptome regulation, however, even residual amounts of m6A may be able to fulfill the functional needs.

#### 2.2.2. P-Bodies

Along with stress granules, the most important cytoplasmic MLOs involved in the regulation of the stress response are Processing-bodies (P-bodies) (Table 1, Figure 1I and Figure 4) [122]. Unlike temporary stress-induced stress granules, P-bodies are constantly present in most of the cell types, and they enlarge and multiply during stress (Figure 4C) [123]. These dynamic compartments are mainly composed of poorly translated mRNA molecules, proteins that contribute to translation inhibition or to different aspects of mRNA degradation, such as 3’-deadenylation, 5’-decapping, 5’-3’ exonuclease activity, and nonsense-mediated decay [124,125]. Additionally, during stress P-bodies, similarly to SG, we incorporate repressed translation initiation complexes, a process that contributes to their enlargement. The marker proteins of these organelles are DDX6, AGO1/3, DCP2, XRN4, EDC3, EIF4E-T, LSM1-7, SMG7, HNRNPM, and CPEB1 [126,127]. A critical role in the formation of P-bodies is played by the phase transitions of helicase scaffold proteins DDX6, EDC-4, LSM-4, and EIF4E-T upon interaction with untranslated mRNA. Inhibition of these proteins causes disassembly of P-bodies [122]. However, the details of the assembly mechanism of mature P-bodies in unstressed cells with low levels of untranslated mRNA remain elusive. It has been established that P-bodies contain hundreds of mRNA types and, probably, in the absence of stress, they serve as a depot for adaptive switching of protein synthesis programs with minimal energy consumption during the cell life cycle [127].

There is strong evidence for functional interplay and cooperation between stress granules and P-bodies (Figure 1H,I and Figure 4C). Two MLOs have been found to share some of the protein and mRNA content, while also having molecules uniquely attributed to one or another. The proteomic analysis showed that the protein composition of stress granules and P-bodies overlaps by 10–25% [128]. Moreover, the composition of stress-induced P-bodies resembles stress granules to an even greater extent [11]. Both SG and P-bodies contain components of the RNA-induced silencing complex (RISC), microRNAs, and argonaute proteins that are needed for RNA interference-induced silencing of mRNA. Additionally, both organelles include RNA-editing enzymes with antiviral activity, such as APOBEC3G (apolipoprotein B mRNA-editing enzyme catalytic subunit 3G) [129]. The presence of so many various catalytically active complexes suggests that these MLOs are the centers for post-transcriptional regulation of gene expression.

SG and P-bodies also were found to directly interact by coming into close spatial proximity that is promoted by regulated molecular tethering (Figure 4C) [125,130]. It has been shown that oxidative stress induced by arsenite promoted the convergence of P-bodies and stress granules and subsequent content exchange in HeLa cells [125]. Two components of mRNA decay machinery TTP and BRF1 were found to promote the SG’s and P-bodies’ physical association [130]. The authors of the study suggested a model of coordinated regulation by SG and P-bodies of mRNA biogenesis during stress. According to this model, firstly, mRNA accumulates to SG for sorting, processing, and storage. Then, mRNA molecules destined for degradation are directly transported into P-bodies via TTP/BRF1 fusions for decay (Figure 4C) [130].

It was also found that, like stress granules, P-bodies can have a multiphase structure in Drosophila oocytes [131]. This type of P-bodies is characterized by two immiscible regions, one containing *gurken* mRNA and the other *bicoid* mRNA. Additionally, in *Drosophila* oocytes, it has been shown that P-bodies and associated U-bodies (MLOs responsible for the assembly and storage of uridine-rich small nuclear ribonucleoproteins that are essential for pre-mRNA splicing) enlarge during starvation [132]. In contrast to mammalian and drosophila cells, yeast P-bodies appear only under stress conditions [133]. Besides that, P-bodies in yeast may be formed under nutrient stress caused by glucose starvation. The resulting bodies are enriched in mRNAs encoding specific mitochondrial oxidative phosphorylation factors such as ATP11, ILM1, MRPL38, and AIM2. At the same time, P-bodies induced by osmotic stresses were depleted by ATP11 [134].

In aging somatic cells of *C. elegans* under stressful conditions, P-bodies regulate proteostasis by recruiting the IFE-2 isoform of the transcription initiation factor eIF4E into these organelles, which contributes to the blocking of protein biosynthesis and increases the lifespan of cells [135].

### 2.3. MLOs Associated with Membrane-Bound Organelles

Dysfunction of cellular homeostasis under stress conditions causes activation of the stress response due to inhibition/activation of specific signaling receptors. As a rule, in eukaryotic cells, these processes occur on the surface of the membranes of cell organelles. The efficient occurrence of this type of reactions often requires the formation of biomolecular condensates on the membrane surface [136]. In this case, the concentrations of proteins required for phase separation are an order of magnitude lower than in the solution. Serine-threonine kinase Target of Rapamycin Complex 1 (TORC1), which is a megadalton complex of four proteins, under normal conditions regulates the synthesis of various biomolecules and inhibits autophagy. The arrest of this receptor activity is accompanied by the formation of TOROID (TORC1 Organized in Inhibited Domain) clusters on the surface of lysosomal membranes. TORC1 reactivation is accompanied by TOROID disassembly [12]. In Drosophila S2 cells, in response to nutrient deficiency, so-called Sec-bodies are formed due to the interaction of the intrinsically disordered protein Sec16 and subunits of the COPII complex [137]. This enables inhibition of protein transport and prevention of damage to vesicle border proteins. Calcium ions are a universal second messenger of various cellular processes that determine cell metabolism [138]. In this regard, under stress conditions, there is a change in the regulation of Ca^2+^-dependent signaling pathways [139]. Catabolic processes observed during the activation of various types of stress responses are regulated by the transport of calcium ions from the ER to mitochondria [139]. An increase in the concentration of calcium ions in mitochondria causes an increase in the production of reactive oxygen species by mitochondria and arrest of the cell cycle, and inhibition of the transport of calcium ions from the ER to mitochondria causes cell death [139]. The so-called MAMs (mitochondria associated membranes) are the platform for calcium transport from the ER to mitochondria [140]. These structures provide the necessary machinery and distance between the ER and the outer mitochondrial membrane for efficient transport of calcium ions. One of the key players in the MAM machinery required for the transport of calcium ions is the family of 1,4,5 triphosphate inositol receptors (IP3R) localized in the ER membrane [140]. In response to an external stimulus, these receptors are activated, which form a complex with the VDAC1 channel localized on the outer mitochondrial membrane and the Grp-75 chaperone, which makes it possible to ensure and coordinate the transport of calcium ions [139]. One of the regulators of IP3R activity, and, accordingly, calcium transport from the ER to mitochondria, is the PML protein [141]. This predominantly nuclear tumor suppressor exists in several isoforms, some of which are capable of cytoplasmic localization [142]. The localization of PML in MAMs is mediated by the cytoplasmic p53 fraction, usually in response to stress [143]. PML is able to form microdomains in MAMs, including IP3R, AKT kinase, and PP2 phosphatase, which ensure phosphorylation and correct operation of IP3R [141]. At the same time, the localization of PML to MAM in the cells of primary mouse fibroblasts contributes to a decrease in autophagy [143]. As is known, MAMs play one of the central roles in the initiation of autophagy, the abundance of MAM is significantly reduced during natural and pathological aging [144]. The key UPR^MT^ stress response receptor, IPE1, also forms clusters in MAMs in response to stress, thereby inhibiting ER-associated mRNA and, accordingly, suppressing the synthesis of new proteins [145]. Endoplasmic reticulum membranes play an extremely important role in the regulation of P-bodies biogenesis. It has been shown that the interaction of endoplasmic reticulum membranes with P-bodies regulates their composition and functional activity [146]. In addition, ER membranes are a platform for the fusion of stress granules and P-bodies.

### 2.4. Yeast MLOs

Yeast cells contain both MLOs that have clear analogues in other eukaryotes, as well as a number of unique yeast-specific condensates (Table 2).

**Table 2:** Examples of LLPS (or suggested to be LLPS) compartments formed or rearranged in response to in yeast cells.

MLO-Type	Main Components	Stress Factors	Structural Changes in Response to Stress	Main Functions
Stress granules	mRNA, Pub1, Pbp1, eIF4GII	Impaired proteostasis, genotoxic stress, temperature, UV irradiation, nutrient deprivation, hypoxia, viral infection, etc.	Assembly of gel-like structures in the cytoplasm.	Storage of capped and polyadenylated mRNAs and their protection from degradation in P-bodies. Regulation of TORC1 signaling
P-bodies	mRNA, Dcp2p and Pat1p [147]	Nutrient deprivation, oxidative and osmotic stress	Assembly of liquid droplets in the cytoplasm. Yeast P-bodies mRNA and proteins composition depends on the type of stress.	Translation repression and mRNA turnover: 3′-deadenylation, 5′-decapping, 5′-3′ exonuclease activity, nonsense-mediated decay
eIF2B bodies	eIF2B	Glucose deprivation	Formation of eIF2B bodies as a result of eIF2B accumulation in the cytoplasm [147].	Involved in inhibition of translation initiation
Proteasome storage granules	Proteasome 19S and 20S subunits [147]	Glucose deprivation	Relocalization of proteasome subunits and formation of proteasome storage granules in the cytoplasm.	Storage of proteasome subunits

Yeast stress granules, in contrast to mammals and Drosophila, exhibit the gel-like properties [110]. Their formation occurs in several stages and is coordinated. In the first step, RNA and RNA-binding proteins interact to form large ribonucleoprotein complexes (RNP complexes). Further, RNP complexes fuse into larger compartments through additional RNA-mediated interactions and, above all, through the binding of prion-like domains. As a result, a solid core is formed, surrounded by a liquid shell [137,148]. At present, the molecular mechanism of SG assembly in fission yeast is not completely clear. It is known that their formation does not depend on the phosphorylation of eIF2α, and glucose starvation-induced yeast SGs lack 40S ribosomal subunits and eIF3, which is a characteristic component and is required for mammalian SG assembly [149]. Yeast has fewer eIF3 subunits than mammals, whereas mammalian eIF4G has an eIF3-binding domain not found in yeast. Therefore, the assembly of yeast SGs is independent of the eIF4G/eIF3 interaction. Multicellular animals have several eIF2α kinases, whereas budding yeasts have only one that also affects the assembly mechanism of SG [150]. It is known that stress granules in the yeast *Schizosaccharomyces pombe* and *Saccharomyces cerevisiae* contain orthologues of proteins found in mammalian SG. In particular, Nxt3, Ubp3, Pub1, PbP1 proteins, orthologues of G3BP, USP10, TIA-1, and Ataxin-2, respectively, were identified. Under heat stress, like their human orthologues, Nxt3 and Ubp3 interact with the RNA-binding protein Pabp and are involved in the formation of stress granules. However, unlike G3BP1 and USP10, neither deletion nor overexpression of nxt3(+) or ubp3(+) affect SG assembly in yeast. Similar results were observed in mutants defective in ataxia-2 and TIA-like proteins, which are important components of SG [151].

Yeast P-bodies are formed independently of stress granules, however, they still contribute to their occurrence. Additionally, when translation is inhibited by glucose deprivation, P-bodies are formed first, then Pab1 accumulates in association with P-bodies, and stress granules appear last [152]. Yeast P-bodies contain the proteins Dcp1p, Dcp2p, Edc3p, Dhh1p, Pat1p, Lsm1p, Xrn1p, Ccr4p, and Pop2p. Studies of yeast P-bodies show that there are clear dependencies in the assembly of specific components. For example, recruitment of Dcp1p to P-bodies is mediated by Dcp2p. The second clear relationship is that Pat1p is required to recruit the Lsm1-7p complex [153]. However, in yeast, deletion of any of the genes encoding P-body components does not compromise their integrity, indicating that they are redundant and cooperative [154]. It has been established that in yeast cells, P-bodies are visible only upon induction of stress [155] and have the properties of liquid droplets since they are soluble by 1,6-hexanediol. P-bodies are heterogeneous in mRNA and proteins depending on the type of stress. Study [155] identified RNAs in yeast P-bodies induced by 10 min glucose fasting or osmotic stress using high concentrations of CaCl2 and NaCl. A total of 1544 glucose starvation mRNAs were present in P-bodies, and 35% of them were stress specific [155]. An analysis of RNA length showed that P-bodies induced by glucose starvation contained shorter RNAs compared to the total pool of activated mRNAs under the corresponding stress conditions, whereas P-bodies induced by osmotic stress contained longer RNAs. This indicates that, at least in yeast, transcript length may be important for P-body recruitment. 

Nutrient stress induces the formation of cytoplasmic aggregates called eIF2B bodies. These MLOs contain subunits of the eIF2B and eIF2 protein complexes and are induced during stress caused by glucose deprivation [147]. One of the major control points in translation initiation involves the activation of eukaryotic initiation factor 2 (eIF2) by eIF2B. eIF2, in its active GTP-bound form, interacts with methionyl-tRNA to form a ternary complex (TC). In yeast, this TC can be associated with initiation factors eIF1, eIF3, and eIF5 to form a multifactorial complex (MFC). The MFC recruits the 40S ribosomal subunit to the mRNA to enable further translation. eIF2B is required for converting eIF2 into a translationally active form. Thus, the eIF2B-dependent response is a highly regulated step in the translation initiation pathway. As a result of stress, phosphorylation of eIF2α by Gcn2p kinase occurs, which leads to a decrease in the cellular pool of active eIF2-GTP and, consequently, to a decrease in the rate of translation initiation. As a result, eIF2B accumulates in the cytoplasm and combines into eIF2B bodies [147]. Yeast eIF2B bodies occur in less than 10% of cells under normal conditions in the logarithmic growth phase but are rapidly induced by stress caused by glucose deprivation. It is important to note that the emergence of eIF2B bodies does not depend on the formation of stress granules. eIF2B bodies are dynamic structures that form faster than stress granules but disassemble more slowly depending on the presence of glucose.

The 26S proteasome is responsible for the proteolysis of a large number of proteins, including important cell cycle regulators. The 26S proteasome cleaves polyubiquitylated substrates in an ATP-dependent manner and can also degrade specific non-ubiquitylated target proteins. In growing and dividing yeast, proteasomes are assembled both in the nucleus and in the cytoplasm. During the transition of the cell to a state of rest or starvation for glucose, the proteasome subunits form large cytoplasmic proteasome storage granules. These granules function as a kind of "reservoir" that stores proteasome subunits until glucose appears in the medium [156].

Acidification of yeast intracellular milieu induces the formation of reversible fibril-like structures [157]. It was shown for IDPR proteins Cdc-19 kinase [158] and glutamine synthetase Gln1 [159]. Regulation of amyloid formation of ATP-producing Cdc-19 yeast kinase are considered as a possible indirect mechanism of SG disassembly. In stress conditions, Cdc-19 fibrillation blocks ATP production. According to [160], after stress glycolytic metabolite fructose-1,6-bisphosphate initiates recruitment of chaperones to Cdc-19 fibrils and promotes solubilization of this kinase. In turn, this causes the synthesis of ATP, a metabolite necessary for the disassembly of stress granules.

## 3. Prokaryotes

Bacteria in nature demonstrate remarkable stress resistance. This is an essential property for survival as the majority of prokaryotic organisms inhabit areas with rapidly and unpredictably changing environmental conditions, such as temperature, pH, salt, oxidation, nutrition, water, and chemical elements availability [161]. In addition to that, prokaryotes invading multicellular organisms must overcome host-defense systems. For example, *E. coli* bacterium upon infection faces bile salts, gastric acid with pH ranging from 2.5 to 4.5, and gastrointestinal tract organic acids [161]. Development of proper adaptation mechanisms and quick responses to various stressors were certainly a great evolutionary requirement that pushed bacteria to evolve complex regulatory networks able to quickly sense dangerous changes in their surroundings and rapidly respond with differential expression of a plethora of regulatory genes. Several major bacterial stress responses have been described, including the general stress response regulated in *E. coli* by sigma38 (rpoS) protein [162], envelope stress response modulated in *E.coli* by sigma(E) factor [163], the heat shock response [164] regulated in *E.coli* by sigma factor-32 (σ32), and the cold shock response regulated by cold-shock proteins [165]. Unlike eukaryotic cells, prokaryotes lack any membrane organelles and the formation of LLPS-driven condensates is a very potent mechanism to substitute for the absence of membrane organelles and spatiotemporally organize thousands of stress factors in a bacterial cytoplasm [166,167,168]. This hypothesis found confirmation in multiple works reporting LLPS-driven cellular moieties in prokaryotes. Just a couple of examples are RNA polymerase clusters (RNAP) in *E. coli* [169], the ParABS protein system responsible for the segregation of bacterial plasmids and chromosomes during proliferation [170], PopZ microdomains, and SpmX condensates in *Caulobacter crescentus* [171,172] and many others.

LLPS condensates are highly responsive to environmental changes making them perfect tools to navigate stress response mechanisms. Similar to eukaryotes, prokaryotic cells were found to both assemble temporal specialized stress-induced membrane-less organelles (such as BR bodies) and rearrange existing condensates in order to combat stress (for instance, SSB and Dps condensates) (Table 3).

**Table 3:** Examples of LLPS (or suggested to be LLPS) compartments formed or rearranged in response to in prokaryotic cells.

Stress-Linked Organelle	Scaffolding Component	Organism	Structural Changes in Response to Stress	Function
SSB condensates	Single-stranded DNA-binding protein (SSB)	*Escherichia coli*	Disassembled in response to stress that causes DNA damage and accumulation of ssDNA.	Serve as storage capsules for SSB protein and other DNA repairing enzymes.
Dps condensates	Dps (DNA-binding protein from starved cells)	*Escherichia coli*	Transform into denser structures in response to stress.	Compact nucleoid during stress conditions, while preserving transcription of genes.
BR bodies (containing RNase E)	RNase E endonuclease	*Caulobacter crescentus, Sinorhizobium meli-loti, Agrobacterium tumefacienes, Escherichia coli, and Cyanobacteria*	Assembled in bacterial cytoplasm in response to stress.	Isolation of untranslated mRNA during stress. Centers for mRNA decay and degradation.
BR bodies (containing RNase Y) [173]	RNase Y endonuclease	*Bacillus subtilis*	Assembled in bacterial cytoplasm in response to stress.	Isolation of untranslated mRNA during stress. Centers for mRNA decay and degradation.
BR bodies (containing RNase J) [174]	RNase J endonuclease	*Helicobacter pylori*	Assembled in bacterial cytoplasm in response to stress.	Isolation of untranslated mRNA during stress. Centers for mRNA decay and degradation.
Granular bodies	IbpA heat shock protein	*Acholeplasma laidlawii*	Assembled in response to stress.	Regulation of heat shock response.
PolyP granules [175]	polyphosphate (polyP)	*Pseudomonas aeruginosa*	Assembled under nitrogen starvation.	Regulation of bacterial cell cycle exit during starvation survival response.

**Figure 5:**
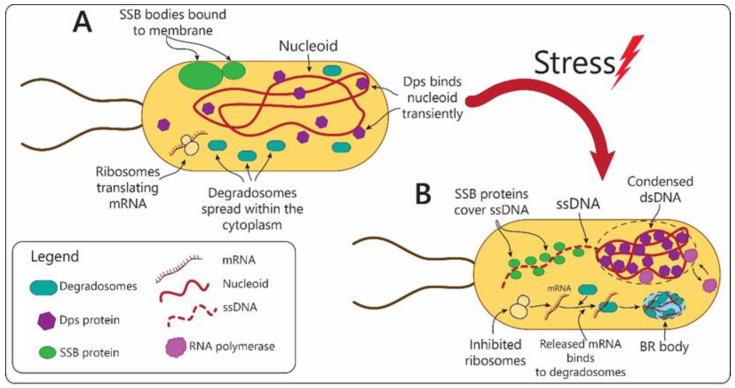
Illustration of changes happening in SSB condensates, Dps condensates, and BR bodies formed by RNaseE degradosomes during stress response in prokaryotes. (**A**) Illustration of unstressed bacterial cell. SSB proteins are sequestered into droplet-like SSB condensates bound to membrane. Dps protein before stress transiently binds to bacterial genome but unable to reach sufficient concentrations to trigger condensate assembly. RNase E tetramers bound to protein partners (degradosomes) are spread throughout the cytoplasm. RNA transcription and translation proceed normally. (**B**) Illustration of cell upon the onset of stress. SSB condensates are disintegrated upon accumulation of single-stranded DNA (ssDNA) and free SSB complexes bind to ssDNA protecting it. Dps complexes heavily cover nucleoid driving the formation of phase separated organelle, which remains permeable for RNA polymerase enzyme. Stress leads to inhibition of translation and release of mRNA from polyribosomes. Untranslated mRNA binds to degradosomes complexes leading to phase separation and assembly of BR bodies.

One instance of rearrangement of pre-existing structures in response to stress can be SSB condensates. Single-stranded DNA-binding proteins (SSB) play vital role in cellular metabolism and survival by binding single-stranded DNA, forming DNA-protein filaments, and preventing potential harmful interactions during DNA replication and DNA damage response (Figure 5A). SSB proteins were found to be present in much larger numbers that are necessary to protect the replication fork during normal DNA duplication [176]. In *E. coli* excess, the SSB protein is stored in a form of structures resembling droplets bound to bacterial membrane, which are rapidly (within 5 min) disassembled upon DNA damage releasing SSB into the bacterial cytoplasm (Figure 5B) [176]. Another study demonstrated that SSB from *E. coli* forms LLPS condensates at physiological conditions in vitro via its intrinsically disordered linker and these biological condensates are quickly disintegrated upon presence of ssDNA [177]. In addition to SSB protein itself, SSB condensates also sequester multiple DNA damage response factors binding to SSB mainly via its C-terminal peptide and are released from the droplets upon DNA damage stress combined with SSB [177]. This mechanism ensures a quick reaction to a highly dangerous condition of single-stranded DNA accumulation with release of necessary DNA reparation machinery ‘ready-to-go’ and active, preserving the precious time and resources for the time-consuming process of protein synthesis and post-translational modification (Figure 5B).

Another DNA-binding protein Dps (DNA-binding protein from starved cells) carrying a DNA-protecting function in *E. coli* also undergoes significant structural rearrangements in response to stress. During the stationary phase in *E. coli* bacteria, Dps massively but transiently binds to nucleoid compacting it (Figure 5A). However, upon serious stress, such as starvation, heat shock, and oxidative stress, Dps heavily covers the nucleoid that leads to formation of condensates, which were proposed to be liquid-liquid phase separated organelles (Figure 5B) [178]. This process is probably driven by the intrinsically disordered N-terminal region of Dps, which has been demonstrated to be essential for Dps DNA-binding activity [179]. Interestingly, the Dps-formed condensates remain permeable for RNA polymerase enzyme, whereas other DNA-binding proteins are excluded, enabling active gene transcription while preventing destruction of the genome.

A certain degree of analogy could be drawn between cytoplasmic stress granules (SG) in eukaryotes and bacterial RNP bodies (BR-bodies). BR bodies are formed as a result of liquid-liquid phase separation of RNaseE endonuclease tetramers called degradosomes (Figure 5A) [180]. The intrinsically disordered C-terminal domain of RNase E facilitates its LLPS transition while multiple protein-partner and RNA binding domains recruit other proteins required for mRNA processing (RNA chaperons, DEAD-box helicases, etc.) as well as RNA molecules [180]. Many microorganisms were found to contain BR bodies, for example, *Caulobacter crescentus, Sinorhizobium meli-loti*, *Agrobacterium tumefacienes*, *E. coli*, and *Cyanobacteria* [166], all of these bacteria species encode RNase E protein. Additionally, in Bacillus subtilis [173] and Helicobacter pylori [174], entities similar to BR bodies were found, but they were formed by different types of endonucleases. 

Similarly to SGs, BR bodies form as a result of the accumulation of free mRNA in the cytoplasm, which is released from the polyribosomes as a result of stress-induced inhibition of translation. Degradosomes interact with untranslated mRNA, a process that drives assembly of BR-bodies (Figure 5B) [180,181]. Although the complete set of BR bodies functional properties is yet to be uncovered, BR bodies are known to modulate mRNA decay and degradation in *E. coli* and *C. crescentus* bacteria [182,183]. Additionally, these condensates demonstrate selective permeability against highly structured RNA molecules, such as rRNA and tRNA, preventing their incorporation into the organelles and, therefore, isolating from the mRNA molecules [182].

Another example of membrane-less organelle formed transiently and only during the stress response is the so-called granular body found in mycoplasma *Acholeplasma laidlawii* [184,185]. *A. laidlawii* granular bodies form in response to heat shock and contain a small heat shock protein, IbpA, which has a subset of interacting partners [186] and assembles into globular-type oligomers and fibrils [187]. *A. laidlawii* belongs to Mollicutes, a class of microorganisms that possess the smallest known genome sizes among autonomously replicating organisms [188] and, thus, developed highly evolutionary optimized gene regulatory networks and metabolic pathways. Having biomolecular condensate-like structure formation as a first-line response towards unfavorable conditions suggests the universal biological significance of membrane-less organelles for cellular survival during stress.

## 4. Factors Regulating Reorganization of MLOs in Stress Response

The main advantage of MLOs that allows such structures to regulate signaling pathways, compared to "classical" organelles, is a fast and reversible response to external stimuli [7]. This is due to the fact that the condensates formed as a result of LLPS of IDPs and other conformationally heterogeneous polymers are metastable structures. Accordingly, a slight change in external conditions can cause a change in the state of such a system and lead to a change in the physical properties of the condensate. The scaffold proteins of the vast majority of MLOs are IDPs and proteins containing IDPRs [5]. The transition of these proteins to the liquid-drop state may be due to a change in the network of their inter- and intramolecular interactions [7]. This can be caused either by a change in the physical characteristics of the environment or by a change in conditionally “biological” factors: post-translational modifications, changes in the concentration of scaffold proteins, and interactions with partners which mostly does not require de novo protein synthesis (i.e., transcription, translation).

### 4.1. “Physical” Factors

Stress conditions are accompanied by changes in the intracellular space of temperature, pH, ionic strength of the solution, osmotic pressure, concentration of metabolites, and reactive oxygen species [1]. Often, a change in one of the physical parameters of the system entails a change in several more. So, heat shock in yeast cells and drosophila causes acidification of the cytoplasmic space [2]. Osmotic shock causes a change in the concentration of salts in the intracellular space, as a result of which the operation of ion channels changes, which in turn can cause a change in cytoplasmic pH [189]. In the cells of a number of bacteria, osmotic stress causes a decrease in the pH of the cytosol. The lack of nutrients in yeast cells causes a decrease in pH in the cytoplasmic space from 7.4 to 6.0 [190]. Cytosol acidification in mammalian and yeast cells is also associated with impaired ion transport under conditions of metabolite deficiency [2]. During aging and related neurodegenerative diseases, deregulation of the transport of calcium ions from the ER to mitochondria is usually observed [138]. Mitochondria are the key organelles involved in the production of energy and metabolites necessary for the cell, therefore, the dysfunction of these organelles is critical for the cell, causing saturation of the cytoplasm with H^+^ ions, which leads to acidification of the intracellular space. The pH in the cytoplasm of tumor cells is also significantly shifted to a more acidic region compared to the characteristic values of healthy cells [2]. As is known, electrostatic inter/intramolecular interactions are one of the main driving forces contributing to the phase separation of IDPs. Accordingly, a change in pH contributes to a change in the network of such interactions, primarily due to a change in the charge of the side groups of amino acid residues [191]. For example, the phase transitions of most of the proteins that make up the stress granules, including the scaffold proteins G3BP1, Pub1, DDX, are pH dependent [192,193,194]. At the same time, G3BP1 can form condensates in the cell in response to pH acidification. Hypoxic conditions associated with pH acidification cause the formation in the nucleoplasm of a special type of A-bodies, the protein composition of which only corresponds to the composition of A-bodies resulting from heat shock by only 20% [101]. Even a small change in temperature can have a significant effect on the interaction of the phase separation of IDPs, changing the network of interactions of "protein–solvent" [195,196]. Depending on the balance between protein–protein interactions, protein-solvent interactions, and protein conformational entropy, the separation of such systems into phases can occur in different temperature ranges, for example, when lower-critical solution temperature (LCST) and upper-critical solution temperature (UCST) are reached [197]. The same picture can be observed when the salt composition of the solution changes, for example, in osmotic stress conditions [198].

### 4.2. “Biological” Factors

Changes in physical environmental factors have a nonspecific effect on all proteins potentially predisposed to LLPS and do not allow for fine regulation of the properties of MLOs. In this regard, transitions of this type play a significant role only at the initial stages of the formation of MLOs. Nonspecific interactions of scaffold proteins of MLOs with mRNA, lncRNA, and rIGSRNA also play a significant role in initiating the formation of MLOs, but not during their maturation [199]. The main factor regulating maturation, attachment of client proteins, and properties of MLOs are post-translational modifications (PTM) of intrinsically disordered proteins [200]. PTMs allow us to specifically change the conditions necessary for the phase separation of a particular protein, depending on the cellular context [201]. Phosphorylation, acetylation, methylation, SUMOylation, and poly-ADP-ribosylation of a number of scaffold proteins of stress granules, including G3BP1, has a significant effect on the correct assembly and functioning of these organelles [202,203,204,205]. On the other hand, phosphorylation of FUS proteins, TDP-43, can reduce the critical concentrations of these proteins required for their phase separation, which makes it possible to weaken the incorporation of these proteins into stress granules, in turn, inhibiting the degradation of SGs [206,207]. O-linked N-acetylglucosaminylation of the hNRNPA1 protein performs the same function [208]. A change in the profile of SUMOylation and acetylation of PML isoforms in response to stress causes a change in the composition of PML bodies and their physical properties [89,209]. The additional evidence of the PTM role in regulation of MLOs assembly/disassembly process may be the reduction in Huntingtin aggregation in the cytosol and chromatin-associated Huntingtin aggregates in the nucleus by SUMO-targeted ubiquitin ligase, Slx5 [210].

Except for PTM, chaperones and autophagosomal degradation play an important role in regulating the properties of MLOs under stress [211]. Chaperone activity ensures correct disassembly of stress-induced MLOs and prevents their degradation into insoluble toxic aggregates. Thus, the HSPB8/BAG3/HSP70 complex prevents the hardening of stress granules [212].

## 5. Discussion

Stress causes the formation of new intracellular environment. The adaptive reaction of cells to the new intracellular environment proceeds at the following levels of cellular organization: genomic, transcriptional, and translational. The genome reorganization is an extreme and mostly irreversible response of the cells to the stress action which is usually observed under conditions of chronic stress [213]. The genome reorganization often leads to pathological cellular transformations.

Cell survival under “physiological stress” (i.e., under the conditions when cells are principally able to return to pre-stress state) is carried out by rearranging its translational and transcriptional profiles. Such cellular program is primarily aimed to change its expression profile and preserve the necessary biomacromolecules. Membrane-less organelles are essential in these processes. The reorganization of cell compartmentalization in response to stress is a fast reversible and adaptive process, primarily aimed at preventing damage to the genetic and protein material of the cell in an aggressive environment. Apparently, this stage of the stress response is a “fire” reaction of the cell to stress, allowing it to survive until the switching of cellular expression programs occurs. The rapid and reversible formation of biomolecular condensates under stress conditions in eukaryotic and prokaryotic cells makes it possible to temporarily exclude the key “complex” biopolymers that provide cell homeostasis from the intracellular space. The synthesis of these molecules (mRNA, rRNA, transcription elongation and initiation factors, and other proteins) is extremely energy consuming. The simultaneous synthesis of these molecules under conditions of nutritional deficiency after stress action can cause cell death.

However, the function of membrane-less organelles under stress conditions is not limited to the protein and genetic preservation. Membrane-less organelles are primarily biomolecular reactors that ensure the occurrence of various biochemical reactions. A number of key enzymes involved in the metabolism of carbohydrates, amino acids, fatty acids, nucleotides in animal cells, yeast, and bacteria are included in the composition of condensates and are activated in response to stress [12]. Therefore, the reorganization of biomolecular condensates under stress conditions is directly related to the activation and regulation of stress signaling pathways.

Reorganization of biomolecular condensates under stress conditions is a systemic response. Formation and alteration of the properties of nuclear membrane-less organelles correlate with changes in the cellular expression profile. A systematic analysis was shown that perturbations of at least 128 genes cause nucleolar enlargement with subsequent formation of stress granules, an increase in the number of Cajal bodies, and splicing speckles in mammalian cells [214]. In addition, this work established a correlation between an increase in the nucleolus and a decrease in P-bodies, wherein no relationship was found between changes in gene expression and the formation of cytoplasmic stress granules [215].

One of the possible regulators of the reorganization of intracellular condensates in response to stress is a change in the localization of their components. Thus, stress conditions cause translocation into the nucleus of the A-bodies scaffold protein VHL [216]. Additionally, transcription inhibition leads to the structural alterations of the nucleolus resulting in the formation of nucleolar caps containing coilin, PML [16], and PATL1 [217]—scaffold proteins of Cajal bodies, PML and P-bodies under normal conditions.

Apparently, RNA turnover can play the same role. The biogenesis of mRNA, rRNA, rDNA, and lncRNA is one of the main regulators of gene expression [201,218,219,220]. These molecules are the key components of stress-induced organelles formed in the cytoplasm, nucleoplasm, and nucleolus under stress conditions. Regulation of these molecules intracellular composition is carried out by recruiting them into stress granules, nuclear stress-bodies, A-bodies, paraspeckles, nuclear speckles, and other membrane-less organelles. It has recently been shown that the regulation of rRNA processing in the nucleolus is a key step in the Ribosome Biogenesis Stress Response pathway [221], which makes it possible to indirectly regulate the structure of the nucleolus, as well as the level of mRNA in the cytoplasm and the formation of stress granules. Under conditions of severe stress, fragmentation of the nucleolus is observed, which in turn causes the release of ribosomal proteins into the cytoplasm, accompanied by inhibition of Hdm2, accumulation of p53, and subsequent induction of apoptosis (Figure 2C) [222].

The formation of new and reorganization of already existing compartments under stress conditions is associated with a change in their material properties. The gelation of MLOs and even the formation of functional amyloid fibrils by them in response to stress is observed in cells of various kingdoms of life. This is due to the need to limit the dynamics of the exchange of the contents of membrane-less organelles in an aggressive intracellular environment. Intrinsically disordered proteins are key actors of these processes. First of all, this is because the material properties of proteins strongly depend on the properties of the environment [196].

Stress-induced reorganization of intracellular milieu is a conservative process and occurs in a similar way in bacterial, yeast, plant, and animal cells. Biomolecular condensates formed in the cells of these organisms are usually regulated by proteins with similar functions. The accumulated data will allow us to state that this form of reorganization of biopolymers is a universal mechanism of stress response.

## 6. Conclusions

The analysis of literature data presented in this work showed that stress-induced rearrangement of liquid-drop cell compartments is a systemic process that regulates cells stress response at the translational and transcriptional levels. In response to unfavorable conditions, both a stress-responsive reorganization of the existing biomolecular condensates and de novo formation of new membrane-less organelles occur in the intracellular environment of eukaryotes and prokaryotes. The phase separation of biopolymers underlying these changes (reorganization) provides a fast, adequate, adaptive, and controlled cell response to any kind of stress. The cell response to stress illustrates the role of biomolecular condensates formed via LLPS for cell physiology

## Figures and Tables

**Table 1:** Examples of LLPS (or suggested to be LLPS) compartments formed or rearranged in response to stress in eukaryotic cells.

	Stress-Linked Organelle	Main Components	Organism	Structural Changes in Response to Stress	Function
**Nuclear membrane-less organelles**					
MLOs subject to change and rearrangement in response to stress	Nucleolus	Fibrillarin, nucleophosmin, rRNA, snoRNPs, Nop58, etc.	Eukarya	Release of ribosomal proteins, change in the nucleolar proteome. Nucleolar segregation upon DNA damage or rRNA transcription. Nucleolar fragmentation upon inhibition of RNA Pol II transcription or protein kinases. Nucleolar and FC enlargement upon viral infection	Ribosome biogenesis
	Cajal bodies	Coilin, SMN1, snRNA, snoRNA, scaRNAs, etc.	Animals and plants	CBs decrease in number and size in response to starvation. CBs undergo disruption and formation of coilin nucleoplasmic microfoci upon UV-C irradiation, osmotic stress, and heat shock. Fusion of transformed CBs with the nucleolus upon GRV infection in plants	Maturation of snoRNA, snRNA, histone mRNA
	Paraspeckles	lncRNA Neat1, NONO, SFPQ, FUS, etc.	Mammals	Increase in paraspeckles numbers upon different types of stress: hypoxia, temperature, sulforaphane treatment, softening of the cellular substrate, etc.	Storage of RNAs and proteins involved in the transcription regulation and pre-mRNA processing.
	Nuclear speckles	snRNP, SR proteins, lncRNA MALAT1, etc.	Mammals and plants	Enlargement and rounding probably via fusions and reincorporation of splicing factors for temporal storage during stress.	Splicing regulation and storage of proteins
	PML-bodies	PML, SUMO-1, Sp100, etc	Mammals Absent in flies, plants and yeasts	Enlargement and decrease in the content mobility upon oxidative stress induced by H2O2. Degradation or cytoplasmic relocalization of the PML isoforms upon oxidative stress induced by As2O3. Decrease in the number and size of PML bodies upon heat stress, heavy metal addition, and expression of adenovirus E1A.	Regulation of the p53-dependent signaling, DNA damage response, DNA repair, telomere homeostasis
Transient assembly in response to stress	NELF bodies	NELF	Human cells	Stress-induced assembly at PolII-active transcription sites driven by NELF protein dephosphorylation and SUMOylation.	Inhibition of RNA Pol II transcription
	Nuclear stress-bodies	HSF1, HSatIII lncRNA, SAFB, hnRNPM	Primates	Stress-induced formation at sites of HSatIII transcription activated by HSF1 transcription factor.	Protein storage and regulation of mRNA splicing
	A-bodies	rIGSRNA, VHL	Mammals, fungi, insects, plants	Assembly and solidification upon the onset of stress at the sites of rIGSRNA transcription.	Temporal storage of amyloidogenic proteins
**Cytoplasmic membrane-less organelles**					
Assembly	Stress-granules	G3BP1, TIA-1, FUS, hnRNPA1, untranslated mRNA, etc	Eukaryotic cells	Reversible assembly in response to stress as a result of accumulation of translationally repressed mRNA in the cytoplasm.	mRNA storage and triage, regulation of translation
Rearrangement	P-bodies	DDX6, EDC-4, LSM-4, EIF4E-T, poorly translated and untranslated mRNA	Eukaryotic cells	Increase in the number and size of P-bodies under stress conditions.	mRNA translation, processing and degradation

## Data Availability

Not applicable.
